# Bedside Neonatal Intensive Care Unit Surgery- Myth or Reality!

**Published:** 2013-04-01

**Authors:** Shandip Kumar Sinha, Sujoy Neogi

**Affiliations:** * Department of Pediatric Surgery Maulana Azad Medical College and Lok Nayak Hospital, New Delhi, India.; 1 Hamdard Institute of Medical Sciences and Research Jamia Hamdard University, Jamia Nagar, New Delhi, India.

## Abstract

Neonatal transport is associated with complications, more so in sick and unstable neonates who need immediate emergency surgery. To circumvent these problems, surgery in Neonatal intensive care unit (NICU) is proposed for these neonates. This article reviews the literature regarding feasibility of this novel concept and based on the generated evidence, suggest the NICU planners to always include infrastructure for this. Also neonatal surgical team can be developed that could be transported.

## INTRODUCTION

Neonatal surgery in neonatal intensive care unit (NICU) is a novel concept which has the potential to decrease the morbidity, mortality and cost of neonatal care. However, it is still not common because of management (infrastructure, manpower) issues. In this review, we have tried to find and compile the evidence for this in literature and based upon that suggest NICU planners to always include provision of same in their NICU.

## MATERIALS AND METHODS

The review of the literature was performed in November 2012 using the Medical Literature Analysis and Retrieval System Online (U.S. National Library of Medicine’s life science data-base; MEDLINE), and Google© search. The MEDLINE search employed both ‘‘MeSH’’ (Medical Subject Heading) and ‘‘free text’’ protocols. Specifically, the MeSH search was conducted by combining the following terms retrieved from the MeSH browser provided by MEDLINE: Intensive Care Units; Neonatal, Neonatal Intensive Care Units; Newborn Intensive Care Units ; Infant, Newborn, Diseases/surgery. Multiple free-text searches were performed applying singularly or in combination the following terms through all the fields of the records: neonatal intensive care unit, neonatal surgery, Laparotomy, patent ductus arteriosus. The related articles related to these searches generated by MEDLINE were also reviewed. Subsequently, the searches were pooled and all articles dealing with surgical intervention of neonates in NICU were included and duplicates were excluded. The authors individually reviewed all the abstracts of the retrieved studies in order to select the papers that were relevant to the review topic. In addition, the reference lists of the included papers were searched for any missing articles. After reviewing the studies, authors analyzed them for indications, results and authors recommendations regarding feasibility of the surgical procedures in NICU. Based upon these authors have also suggested future course of action for further development of this field.

## RESULTS

Search of text ‘Intensive Care Units, Neonatal, neonatal surgery, Patent ductus arteriosus’ yielded 63 articles of which 18 were included. Search of Text ‘Newborn Intensive Care Units, neonatal surgery, Laparotomy’ yielded 17 articles of which 2 were included. After searching related articles and references from these articles, 26 articles dealing with surgical intervention in neonates in NICU were included in study (Table 1, 2).

**Figure:**
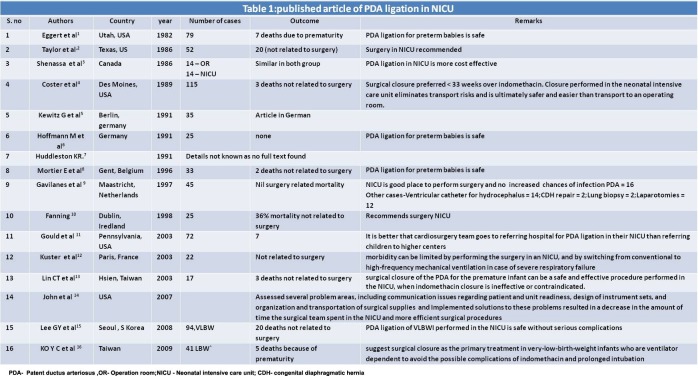
Table 1

**Figure:**
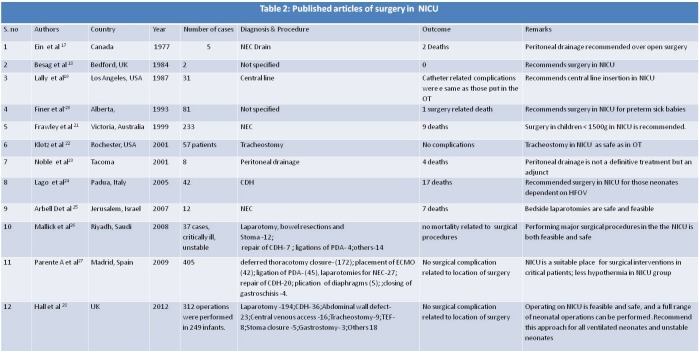
Table 2

## DISCUSSION

Surgery in neonates is required for a number of conditions, in emergency settings (necrotizing enterocolitis etc.), semi emergencies like congenital diaphragmatic hernia (CDH), esophageal atresia and routine cases like hernia. These neonates may be stable or unstable and many of them may be on mechanical ventilator. In non emergent cases, the neonates can be stabilized and then can be taken for surgery. However, in many emergency situations, surgery may be needed immediately. The surgery of these patients involves shifting to operation theater (OT), and then back from OT to Neonatal intensive care unit (NICU). This may mandate inter-hospital or intra-hospital transport. This needs neonatal transport. The adverse effects of inter-hospital transport of neonates are well documented [1, 27]. These include risk of deterioration especially in ventilated and unstable neonates. 

Also, there are country wise differences in organization of neonatal transport units ranging from dedicated transport services to carry out all transfers to adhoc systems provided by big hospitals [28, 29]. This leads to a situation where unstable ventilated neonates are transferred in suboptimal conditions. This is more true for neonates requiring surgery, as all over the world there are stand-alone NICU with no surgical facilities and neonates who need surgery are transferred to centers with facility for neonatal surgery. Intra-hospital transport of critically ill patients is also associated with complications. Although there are no studies in neonates, in an Australian study for critically ill adult patients, serious adverse outcomes including major physiological derangement, patient/relative dissatisfaction, prolonged hospital stay, physical/psychological injury and death were reported [30]. To obviate these problems, operations of sick surgical neonates in NICU are proposed. The neonates can be operated in NICU where surgical facilities are available or a neonatal surgical team could be developed that could be transported for surgery. The major indications for surgery in NICU are the procedures which are needed in neonates on mechanical ventilator or emergently in an unstable patient.

 Most of these neonates are premature, very low birth weight (VLBW), may be on prolonged ventilator support [31] and may need surgery for congenital or acquired conditions. These neonates have high risk of transport related complication. 

However this has problems related to infrastructure, manpower and patient outcome [26]. In this review article, we have aimed to generate evidence in relation to surgery of neonates in NICU rather than OT.

**What neonatal surgeries had been done safely in NICU?**

A review of the literature suggest that the since the publication of abdominal laparotomy drain for NEC in 1977 [15] and ligation of patent ductus arteriosus (PDA) in 1982 [1] in NICU, a plethora of surgeries have been performed in NICU. PDA ligation is the commonest surgery that has been safely performed in NICU. The results of PDA surgery are reviewed in Table 1. Since first reported by Eggert et al [1] in 1982 [16], published studies from all around the world (USA, Netherlands, Germany, Taiwan, South Korea, etc.) confirm that a total of 669 neonates with PDA had successful ligation in NICU without any major complication. NICU PDA ligation has been known to be safe as well as cost effective [1-7]. In fact, it is has been reported better than the medical treatment (Indomethacin) [3, 15]. The pediatric cardiothoracic surgical team goes to NICU and performs the surgery avoiding any shifting of the sick neonate, while maintaining the continuity of care (same neonatologist) [9]. In spite of such a large worldwide experience, NICU PDA ligation has not gained universal acceptance as most of the NICU planners do not incorporate the idea of neonatal surgery in intensive units; even basic surgical instruments and not always available in NICU.

Laparotomy for necrotizing enterocolitis (NEC) is the other commonly reported surgery that has been performed in NICU [19]. Most of these are sick unstable ventilated neonates. Apart from this, CDH repair also has been described (Table 2). Some NICUs have a well-established protocol for operative repair of CDH in NICU [22]. Newborns with CDH symptomatic at birth were sedated and paralyzed in the delivery room, and treated with elective high-frequency oscillatory ventilation (HFOV), surfactant, inhaled nitric oxide (iNO) and membrane oxygenation (ECMO) as necessary, delaying surgical repair until their clinical conditions were stable. Once the CDH newborn was stabilized, a trial on conventional ventilation was started at least 24 hours before surgery; however, if the patient was unstable, therapy was switched back to HFOV and surgery was performed in the NICU. They recommended a prolonged phase of pre-surgery stabilization and strict control of infection for the CDH newborns that might benefit from an exclusive HFOV and NICU surgery.

Besides, tracheostomy [20], central line placement [17], tracheoesophageal fistula (TEF) repair [26], abdominal wall defect repair [32], stoma closure [26], gastrostomy [26] have been also done safely in NICU (Table 2). A list of surgeries that could be performed in NICU has been tabulated (Table 3).

**Figure:**
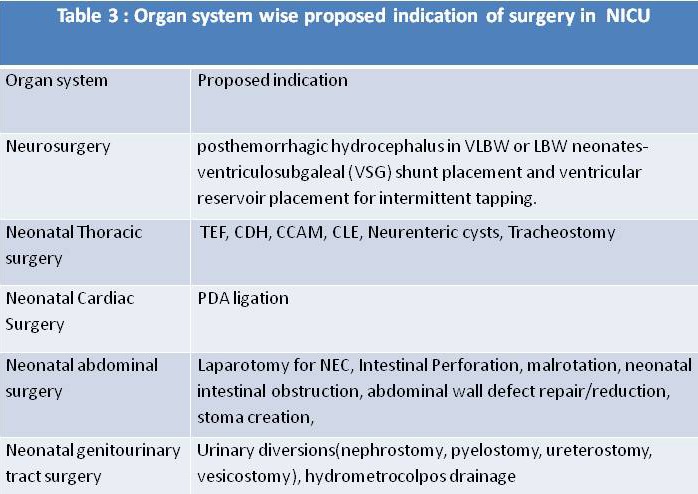
Table 3

**What are the infrastructures needed for surgery in NICU?**

Almost all the NICU have certain basic instruments which are routinely used. These non consumable instruments can be used for surgery also (Table 4). As apparent from the table, very few extra instruments/ infrastructural changes are needed to perform surgery in NICU. All future NICU planners must integrate OT, equipped with the common surgical consumables in NICU. 

**Figure:**
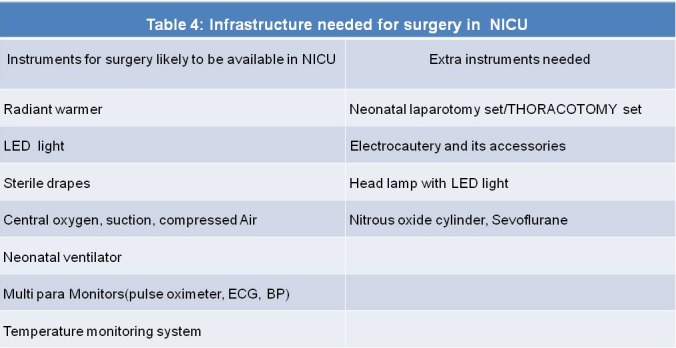
Table 4

**What are the members needed for NICU operative team? **

Every hospital should have a dedicated team to undertake these procedures. A sample team can consist of senior neonatal surgeon, two neonatal surgeons as assistants (one may be trainee), two trained surgical nurses (one scrub nurse and the other floor nurse), one technician to maintain the instruments and two neonatal anesthetists. Each neonatal NICU should have dedicated cupboard for surgical supplies that should be replenished after each surgery. 

According to British Association of Perinatal Medicine Standards for Hospitals Providing Neonatal Intensive and High Dependency Care and Categories of Babies requiring Neonatal Care guidelines, level III Units should provide the whole range of medical neonatal care but not necessarily all specialist services such as neonatal surgery [33]. However, they recommend defined lines of communication and access to specialist advice including Neonatal Surgery and Anaesthesia. As neonatal surgery is required only a few of the neonates, this policy of neonatal ICU operative team covering designated level III NICU within a geographical area can be utilized for optimal utilization and greater reach of resources. It has the advantages of maintaining the continuity of care and minimal inconvenience to mother. With more and more level II and level III NICU being developed all over the world, this policy can solve human resource problems for neonatal surgery.

## CONCLUSION

Neonatal surgery in NICU is a safe procedure and can be utilized in unstable or ventilated neonate. Every neonatal ICU planner should always create infrastructure for surgery in NICU. Surgical NICU operative team should be developed for optimal reach and utilization of resources.

## Footnotes

**Source of Support:** Nil

**Conflict of Interest:** None

